# Anandamide Concentration-Dependently Modulates Toll-Like Receptor 3 Agonism or UVB-Induced Inflammatory Response of Human Corneal Epithelial Cells

**DOI:** 10.3390/ijms22157776

**Published:** 2021-07-21

**Authors:** Ágnes Angyal, Zsófia Pénzes, Shahrzad Alimohammadi, Dorottya Horváth, Lili Takács, György Vereb, Barbara Zsebik, Tamás Bíró, Kinga Fanni Tóth, Erika Lisztes, Balázs István Tóth, Attila Oláh, Attila Gábor Szöllősi

**Affiliations:** 1 Department of Physiology, Faculty of Medicine, University of Debrecen, Egyetem tér 1, 4032 Debrecen, Hungary; angyal.agnes@med.unideb.hu (Á.A.); toth.kinga.fanni@med.unideb.hu (K.F.T.); lisztes.erika@med.unideb.hu (E.L.); toth.istvan@med.unideb.hu (B.I.T.); olah.attila@med.unideb.hu (A.O.); 2 Doctoral School of Molecular Medicine, University of Debrecen, Egyetem tér 1, 4032 Debrecen, Hungary; penzes.zsofia@med.unideb.hu (Z.P.); shahrzad.alimohammadi@med.unideb.hu (S.A.); horvath.dorottya@med.unideb.hu (D.H.); 3 Department of Immunology, Faculty of Medicine, University of Debrecen, Egyetem tér 1, 4032 Debrecen, Hungary; biro.lcmp@gmail.com; 4 Department of Ophthalmology, Faculty of Medicine, University of Debrecen, Egyetem tér 1, 4032 Debrecen, Hungary; ltakacs@med.unideb.hu; 5 Department of Biophysics and Cell Biology, Faculty of Medicine, University of Debrecen, Egyetem tér 1, 4032 Debrecen, Hungary; gvereb2020@gmail.com (G.V.); zsebik.barbara@pharm.unideb.hu (B.Z.); 6 MTA-DE Cell Biology and Signaling Research Group, Faculty of Medicine, University of Debrecen, Egyetem tér 1, 4032 Debrecen, Hungary; 7 Faculty of Pharmacy, University of Debrecen, Egyetem tér 1, 4032 Debrecen, Hungary; 8 Monasterium Laboratory Skin & Hair Research Solutions, Mendelstraße 17, 48149 Münster, Germany

**Keywords:** endocannabinoid, inflammation, anandamide, TLR3, cornea

## Abstract

Photodamage-induced and viral keratitis could benefit from treatment with novel nonsteroid anti-inflammatory agents. Therefore, we determined whether human corneal epithelial cells (HCECs) express members of the endocannabinoid system (ECS), and examined how the endocannabinoid anandamide (AEA, N-arachidonoyl ethanolamine) influences the Toll-like receptor 3 (TLR3) agonism- or UVB irradiation-induced inflammatory response of these cells. Other than confirming the presence of cannabinoid receptors, we show that endocannabinoid synthesizing and catabolizing enzymes are also expressed in HCECs in vitro, as well as in the epithelial layer of the human cornea in situ, proving that they are one possible source of endocannabinoids. p(I:C) and UVB irradiation was effective in promoting the transcription and secretion of inflammatory cytokines. Surprisingly, when applied alone in 100 nM and 10 μM, AEA also resulted in increased pro-inflammatory cytokine production. Importantly, AEA further increased levels of these cytokines in the UVB model, whereas its lower concentration partially prevented the transcriptional effect of p(I:C), while not decreasing the p(I:C)-induced cytokine release. HCECs express the enzymatic machinery required to produce endocannabinoids both in vitro and in situ. Moreover, our data show that, despite earlier reports about the anti-inflammatory potential of AEA in murine cornea, its effects on the immune phenotype of human corneal epithelium may be more complex and context dependent.

## 1. Introduction

Corneal epithelial cells, which make up the outermost layer of the cornea and protect the inner layers from pathogens and physical challenges, are responsible for forming both a physical and an immunological barrier. Damage to the corneal epithelium results in inflammation, which can lead to either restitution of the barrier or, if the damage is more extensive, a loss of transparency [1]. Although corneal reepithelization is an intensively studied area of experimental ophthalmology [2,3,4], better understanding of corneal inflammation is still an important challenge in the field.

An important contributor to corneal inflammation is the transient receptor potential vanilloid 1 (TRPV1) ion channel, which has been reported to be expressed by both trigeminal nerve endings in the cornea [5] and corneal epithelial cells, where the activation of the channel might exacerbate corneal inflammatory processes [6]. TRPV1 is a calcium-permeable non-specific cation channel that can be activated by a wide range of internal and external factors [7], and is considered to be a central integrator of painful and inflammatory stimuli [8]. Activation of the channel on corneal epithelial cells results in pro-inflammatory cytokine and chemokine expression [6]. The importance of TRPV1 in corneal inflammation and wound healing is highlighted by the finding that, in Trpv1^-/-^ animals, inflammation in the corneal alkali burn model is suppressed, and the development of corneal fibrosis is inhibited [9]. As such, TRPV1 is considered a possible target to decrease excessive ocular inflammatory processes. Indeed, resiniferatoxin, an ultrapotent activator of TRPV1 [10], can be used as an analgesic agent, since topical application of this vanilloid leads to prolonged analgesia without affecting non-pain sensing fibers, or causing cytotoxicity in TRPV1-positive corneal epithelial cells [11].

Another promising avenue of novel anti-inflammatory and analgesic treatments is cannabinoids, which have been reported to have considerable effects on multiple cell types [12,13]. The realization that *Cannabis* preparations cause corneal analgesia forms the basis of the Gayer test [14], which was historically used to test the potency of various marijuana extracts [15,16]. A synthetic cannabinoid receptor type 1 (CB_1_) agonist was subsequently shown to affect corneal nerve fibers [17], but its effects on non-neuronal structures were not evaluated at the time. A later study found that CB_1_ is also expressed on human corneal epithelial cells (HCECs), where it is functionally linked to TRPV1 [18]. Indeed, CB_1_ activation by the synthetic agonist WIN55, 212-2 and the endogenous agonist N-arachidonoylethanolamine (anandamide; AEA) resulted in an increased intracellular calcium concentration, most likely via the indirect activation of TRPV1, since the effect can be blocked by the TRPV1 antagonist capsazepine [18]. Although this observation suggests that, similar to epidermal keratinocytes [19], biological activities of CB_1_ and TRPV1 are coupled together in certain cases, other data argue that they may also play differential roles in corneal epithelial cells. Indeed, the selective TRPV1 activator capsaicin was found to promote the release of pro-inflammatory cytokines in these cells. Importantly, this effect was prevented by AEA in a CB_1_-dependent manner, since co-administration of the CB_1_ antagonist AM251 abrogated the anti-inflammatory action of AEA [18]. Considering that, together with other endocannabinoids and structurally related compounds, AEA was already shown to be present in human corneal tissue [20], these results suggested that AEA-dependent CB_1_ signaling may exert a constitutive anti-inflammatory effect in this compartment of the eye [18]. 

Importantly, however, the exact source of endocannabinoids in relation to corneal cells has not been investigated to date. The fact that, under physiological circumstances, the cornea is an avascular tissue, points to the possibility that corneal epithelial cells may not rely on the uptake of circulating endocannabinoids but might rather be capable of producing endocannabinoids on their own. Thus, we first aimed to identify whether corneal epithelial cells could be the source of the two most common endocannabinoids, i.e., AEA and 2-arachidonoylglycerol (2-AG), by investigating the expression of the major enzymes required for the production (AEA: N-acyl phosphatidylethanolamine-specific phospholipase D [NAPE-PLD]; 2-AG: diacylglycerol lipase [DAGL]-α and -β) and degradation (AEA: fatty acid amide hydrolase [FAAH]; 2-AG: monoacylglycerol lipase [MAGL]) of these lipid mediators [21].

Building on these results, in our functional experiments, we wished to assess whether AEA had similar anti-inflammatory effects in the context of other types of inflammation in HCECs. To achieve this goal, we used two distinct types of clinically highly relevant inflammatory stimuli, namely ultraviolet B (UVB) irradiation and Toll-like receptor (TLR)-3 activation (via the synthetic agonist polyinosinic:polycytidylic acid; p(I:C)), to mimic photodamage and viral keratitis, respectively, both of which have been shown to elicit inflammatory mediator production and release in HCECs [22,23,24].

## 2. Results

**Figure 1:**
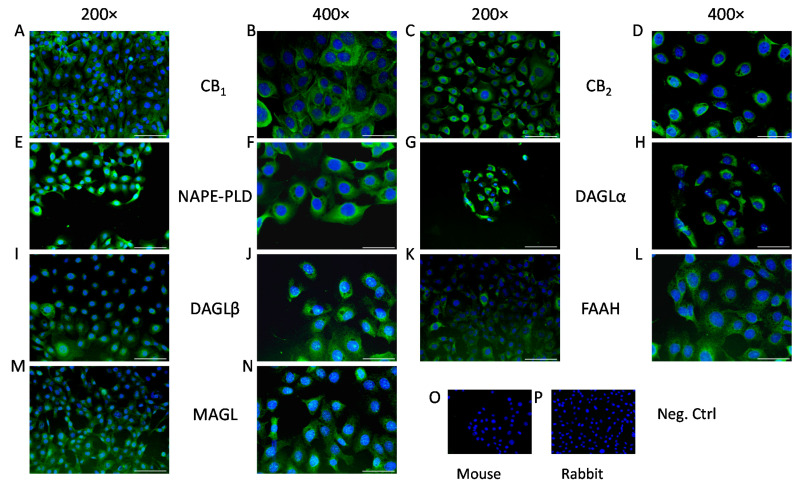
Human corneal epithelial cells express endocannabinoid receptors and endocannabinoid metabolizing enzymes in vitro: (**A**,**B**) CB_1_-specific, (**C**,**D**) CB_2_-specific, (**E**,**F**) NAPE-PLD-specific, (**G**,**H**) DAGLα-specific, (**I**,**J**) DAGLβ-specific, (**K**,**L**) FAAH-specific, and (**M**,**N**) MAGL-specific immunoreactivity, as shown by immunofluorescence (green fluorescence) on human corneal epithelial cell cultures. Nuclei were counterstained with DAPI (4′,6-diamidino-2-phenylindole; blue fluorescence). (**O**) Negative control of panels C, D, G–N; (**P**) negative control of panels A, B, E, and F. Scale bars mark 100 μm or 50 μm, depending on magnification (200× and 400×, respectively).

**Figure 2:**
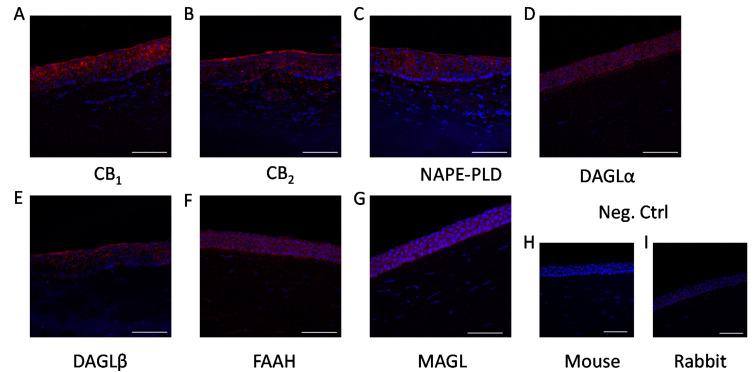
Epithelial cells of the human cornea express endocannabinoid receptors and endocannabinoid metabolizing enzymes in situ: (**A**) CB_1_-specific, (**B**) CB_2_-specific, (**C**) NAPE-PLD-specific, (**D**) DAGLα-specific, (**E**) DAGLβ-specific, (**F**) FAAH-specific, and (**G**) MAGL-specific immunoreactivity, as shown by immunofluorescence (red fluorescence) on human cornea sections. Nuclei were counterstained with DAPI (blue fluorescence). (**H**) negative control of panels **B**,**D**–**G**; (**I**) negative control of panels **A**,**C**. Scale bars mark 50 μm.

As the first step in our experiments, we investigated the presence of the aforementioned enzymatic apparatus and classical cannabinoid receptors in HCECs, both in vitro and in situ. We confirmed that, in line with the already published data, [18,25] HCECs expressed classical cannabinoid receptors (CB_1_ and CB_2_; Figure 1A–D). Moreover, we could also demonstrate that endocannabinoid synthesizing (NAPE-PLD, DAGLα and -β; Figure 1E–J, respectively) and degrading enzymes (FAAH and MAGL; Figure 1K–N, respectively) are also expressed in cultured HCECs, proving that they are one possible source of endocannabinoids in the cornea. Importantly, the same proteins were also found to be expressed in the epithelial cell layer of the human cornea in situ (Figure 2).

**Figure 3:**
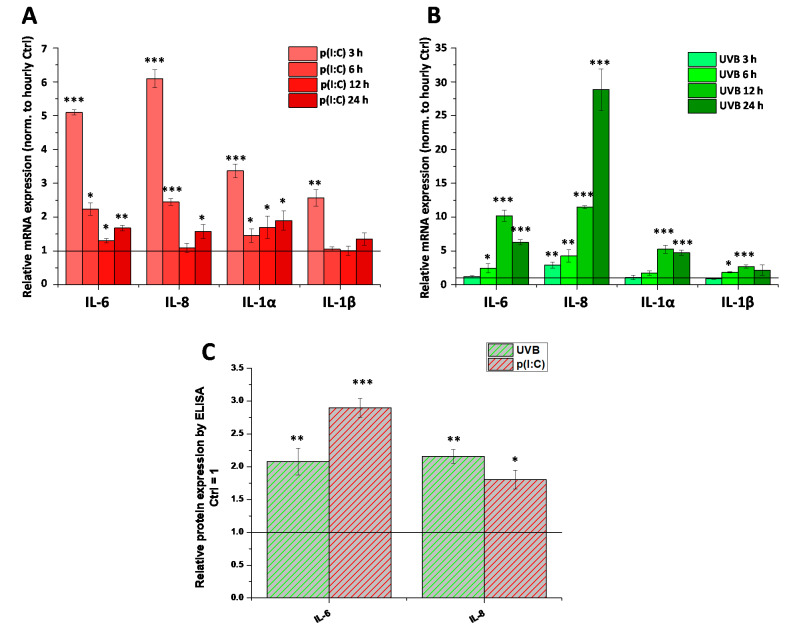
Inflammation can be induced in human corneal epithelial cells by both TLR3 activation and UVB irradiation: (**A**,**B**) Q-PCR of IL-6, IL-8, IL-1α, and IL-1β mRNA from cultured human corneal epithelial cells. Expression was determined following 3, 6, 12, 24 h after p(I:C) treatment (**A**) and 3, 6, 12, 24 and 48 h after UVB irradiation (40 mJ/cm^2^) (**B**). Data are presented using the ΔΔCT method compared to 18SRNA (**A**) and PPIA (**B**), normalized to the expression of the vehicle-treated control (shown as a continuous line at 1). Data are expressed as mean ± SD of 2–3 determinations. One additional experiment yielded similar results. *, **, *** mark significant changes compared to the time-matched control group (*p* < 0.05, 0.01, 0.001, respectively), as indicated. (**C**) ELISA determination of IL-6 and IL-8 in the supernatant of human corneal epithelial cells following 3 h p(I:C) and 12 h after UVB irradiation. Data are expressed as mean ± SD of three determinations. One additional experiment yielded similar results. *, **, *** mark significant (*p* < 0.05, 0.01, 0.001, respectively) changes compared to the vehicle control as 1 (solid line).

**Figure A1:**
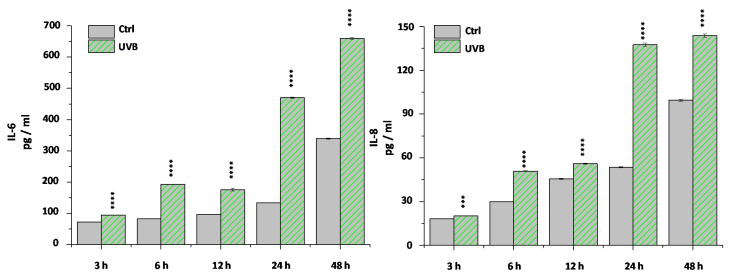
Time course of cytokine secretion after UVB irradiation from HCEC cells. ELISA determination of IL-6 and IL-8 in the supernatant of human corneal epithelial cells following UVB irradiation at the marked time points. Data are expressed as mean ± SD of three independent determinations. ***, **** mark significant (*p* < 0.001, 0.0001, respectively) changes compared to the relevant time-matched control group.

Next, we aimed to test whether the already-described CB_1_-dependent anti-inflammatory effect of AEA [18] is a universal action, i.e., whether it develops in case of non-TRPV1-mediated inflammatory stimuli. To this end, we first examined the ability of two separate, clinically highly relevant inflammatory initiators, the TLR3 ligand p(I:C) and UVB irradiation, to induce inflammatory responses mimicking viral keratitis and photodamage in our HCECs, respectively. We found that both stimuli were effective in promoting the transcription of inflammatory interleukins (interleukin [IL]-6, IL-8, IL-1α, and IL-1β, Figure 3A,B). Both stimuli were tested at multiple time-points (3, 6, 12, and 24 h for both treatments, as well as 48 h for UVB), and subsequent samples were treated only for the time with the most marked changes (3 h for p(I:C) and 12 h for UVB treatment). Changes in mRNA level were validated with ELISA, where we saw that the secretion of IL-6 and IL-8 was increased by both stimuli at the abovementioned timepoints (Figure 3C). Cytokine secretion after UVB irradiation was also determined at multiple timepoints (Figure A1). Neither IL-1α secretion nor IL-1β secretion were detected at any tested timepoint after UV treatment (data not shown).

**Figure 4:**
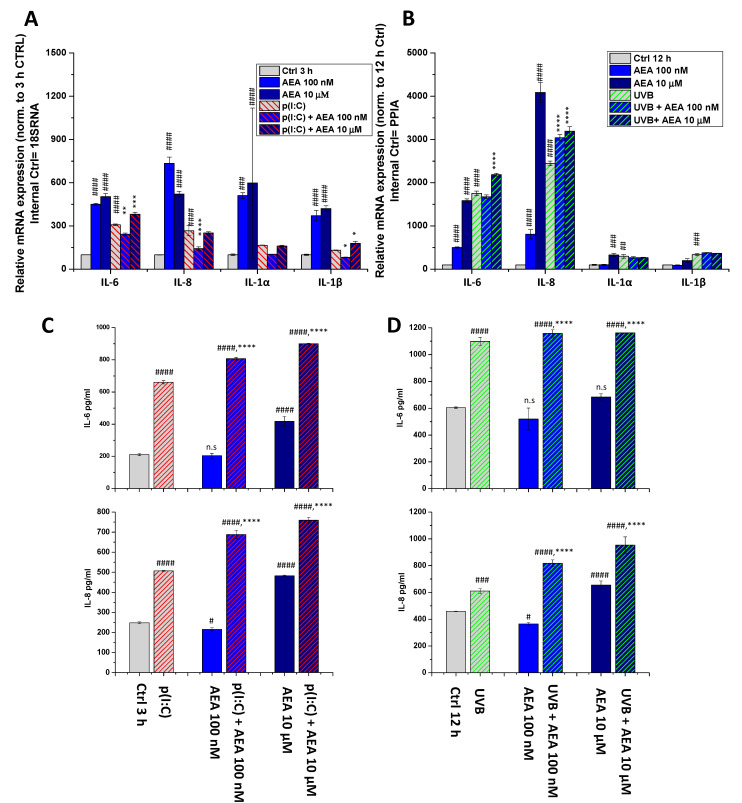
AEA exerts concentration-dependent effects on the inflammatory cytokine expression and release of human corneal epithelial cells: (**A**,**B**) Q-PCR IL-6, IL-8, IL-1α, and IL-1β mRNA expression was determined following 3 h p(I:C) treatment (**A**) and 12 h after 40 mJ/cm^2^ UVB irradiation (**B**). Data are presented using the ΔΔCT method compared to 18SRNA (**A**) and PPIA (**B**), normalized to the expression of the vehicle-treated control (shown as 100). Data are expressed as mean±SD of 2–3 determinations. One additional experiment yielded similar results. *, **, ***, **** mark significant (*p* < 0.05, 0.01, 0.001, 0.0001) differences compared with the UVB- or the p(I:C)-treated group; #, ##, ###, #### mark significant (*p* < 0.05, 0.01, 0.001, 0.0001) differences compared to the vehicle-treated control. (**C**,**D**) ELISA determination of IL-6 and IL-8 in the supernatant of human corneal epithelial cells following 3 h p(I:C) and 12 h after UVB irradiation. Data are expressed as mean ± SD of three determinations. One additional experiment yielded similar results. *, **, ***, **** mark significant (*p* < 0.05, 0.01, 0.001, 0.0001, respectively) changes compared to the treated group; #, ##, ###, #### significant (*p* < 0.05, 0.01, 0.001, 0.0001) differences compared to the control.

To assess the potential anti-inflammatory effects of AEA on HCECs, we next tested the effects of its low (100 nM) and high (10 μM) concentrations in the aforementioned models. As a necessary control experiment, we also tested the effect of AEA as a monotreatment (Figure 4). Surprisingly, AEA at both concentrations caused a significant increase in the transcription of all investigated cytokines at 3 h (Figure 4A). This increase was still present at 12 h in the cases of IL-6 and IL-8 after 100 nM AEA treatment, and for all cytokines after 10 μM AEA (Figure 4B). Interestingly, however, at 100 nM, AEA combined with p(I:C) significantly reduced the transcription of all investigated ILs compared to p(I:C) applied alone (3 h treatments). At 10 μM, on the other hand, we observed the opposite effect, i.e., AEA caused an increase in the levels of IL-6 and IL-1β, while having no effect on IL-8 and IL-1α (Figure 4A).

**Figure A2:**
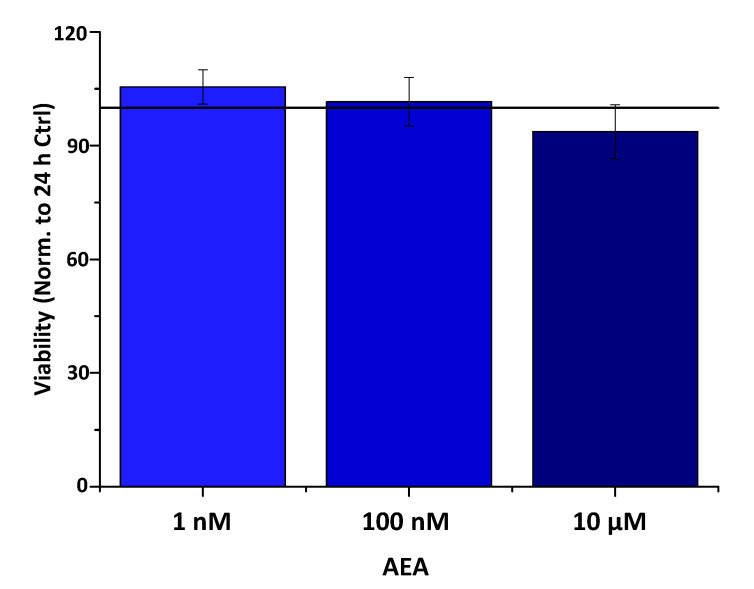
AEA does not influence the viability of HCECs. Determination of cell viability by colorimetric MTT (3-(4,5-dimethylthiazol-2-yl)-2,5-diphenyl tetrazolium bromide) cell viability assay. Data (mean ± SEM) are expressed as a percentage of the mean value (defined as 100%) of the vehicle-treated group. Three additional experiments yielded similar results.

In the case of UVB irradiation, on the other hand, we experienced no decrease subsequent to AEA treatment at any dose. Moreover, 10 μM of AEA increased both IL-6 and IL-8 levels compared to the UVB-treated group, while 10 nM of AEA only increased the latter (Figure 4B). We wished to validate our most prominent findings on the protein level using ELISA, where we found that p(I:C) increased the secretion of both IL-6 and IL-8, while 100 nM of AEA alone had no significant effect on IL-6, while slightly suppresssing IL-8 and 10 μM caused an increase in the secretion of both cytokines. Combining either concentration of AEA with p(I:C) once again caused an additional, significant increase in IL secretion compared to p(I:C) applied alone (Figure 4C). UVB treatment showed similar results; however, only IL-8 secretion was increased after 10 μM of AEA treatment. The combination of UVB and AEA treatment once again caused an additional significant increase in the secretion of both cytokines (Figure 4D). To rule out the role of cell death in the above changes, cellular viability was assessed with an MTT assay, which showed that AEA caused no significant change in cellular viability (Figure A2).

## 3. Discussion

The epithelial cells of the cornea act as the first line of defense for the ocular surface and, as such, they respond both to physical and immunological challenges [1]. The importance of the latter is underlined by the fact that excessive inflammation can lead to the loss of corneal transparency, which is most commonly remedied by a corneal transplant [26]. The direct involvement of HCECs in inflammation is supported by the findings that they are capable of initiating and sustaining inflammation both by the production of inflammatory mediators [27] and also by the presentation of antigens via MHC-II [28].

One possible initiator of inflammatory responses on HCECs is TRPV1, since the activation of this multimodal receptor results in the production and release of inflammatory mediators from these cells [6,18]. Based on these results, TRPV1 antagonists can be considered a possible therapeutic intervention in the treatment of corneal inflammation. Indeed, in a murine allergic conjunctivitis model, TRPV1 antagonists proved to be effective in ameliorating the clinical signs of ocular inflammation [29], although the observed beneficial effects in this work were at the level of T cells, not the corneal epithelium. Unfortunately, it is well-documented that TRPV1 antagonists may have unintended side effects on body temperature, ranging from hyper- to hypothermia [30]. To the best of our knowledge, the sole TRPV1 antagonist specifically tested for ocular use is SYL-1001, which completed phase II clinical trials (ClinicalTrials.gov Identifier: NCT02455999) in 2018; however, no results have been posted to date [31]. Although systemic side effects might not be present in a topical formulation, research has also turned toward other avenues to influence the activation of TRPV1, namely the endocannabinoid system. 

Cannabinoids, in general, have been found to be anti-inflammatory [12] and, in the case of corneal epithelial cells, one of the prototypical endocannabinoids, AEA, has been shown to function upstream of TRPV1 by dampening its activation and the resulting inflammation [18]. Corneal tissue contains two of the most common endocannabinoids, AEA and 2-AG [32] and, in our current work, we were able to show in vitro (Figure 1) and in situ (Figure 2) that HCECs express the enzymatic machinery required for the synthesis and degradation of both AEA (NAPE-PLD and FAAH, respectively) and 2-AG (DAGLα and –β, as well as MAGL), and we also confirmed the presence of the classical cannabinoid receptors CB_1_ and CB_2_. Even though 2-AG has a higher concentration in the cornea compared to AEA [20] (a common finding in most tissues) [33], the role of AEA in corneal wound healing and inflammation has been more extensively investigated [18]. For this reason, in our subsequent functional experiments, we focused on elucidating its role in inflammatory signals that are most likely independent of TRPV1. 

Two common stressors faced by these cells are viral infections [34] and UV light [35], which we modelled by activating TLR3 with p(I:C) treatment and UVB irradiation, respectively (Figure 3 and Figure A1). We found that both stimuli resulted in increased production (IL-6, IL-8, IL-1α for both stimuli, and IL-1β for UVB only) and release (IL-6 and IL-8) of inflammatory cytokines in vitro, while IL-1α and IL-1β secretion were not detected. We next added AEA in combination with these treatments at both low (100 nM) and relatively (yet not unprecedentedly) [18] high (10 μM) concentrations, since a concentration-dependent dual effect has been described in relation to AEA in the presence of TPRV1 [36]. We found that a low concentration of the endocannabinoid was capable of reducing the transcription of inflammatory mediators elicited by p(I:C) only, and the same effect was not apparent with UVB. Surprisingly, higher concentrations of AEA had an opposite effect, in that they caused a further increase in the transcription and release of certain cytokines in the case of both inflammatory stimuli (Figure 4). Another striking finding of our experiments is that AEA applied alone had a marked pro-inflammatory effect, resulting in increased production of cytokines from HCECs (Figure 4).

These results seemingly contradict previous reports in which activation of CB_1_ with a synthetic agonist or AEA inhibited the pro-inflammatory response subsequent to TRPV1 activation [18,37]. In our current models, where the inflammatory conditions were elicited putatively independently of TRPV1 (i.e., via the activation of TLR3 and UVB irradiation), high doses of AEA had a distinctly pro-inflammatory effect on both mRNA and protein levels of cytokine produced by these cells (Figure 4). Interestingly, low AEA concentrations had the same effect on cytokine secretion, even though the transcription of pro-inflammatory cytokines was partially normalized (Figure 4A), suggesting that AEA might differentially modulate the release of the already-existing, pre-synthesized cytokine pool and de novo production of these molecules.

Importantly, AEA is known to activate TRPV1; thus, it is possible that it is only anti-inflammatory if TRPV1 is activated by a more efficacious ligand on corneal epithelium during inflammation. In this context, in the absence of pro-inflammatory TRPV1 signaling, AEA itself becomes pro-inflammatory, and this inflammation may be additive in nature to other inflammatory pathways, such as those caused by UVB and p(I:C). 

Moreover, AEA has also been described to act on many other targets including, but not limited to GPR55 [38], GPR18 [39], Ca_V_3.1, Ca_V_3.2, Ca_V_3.3 [40], K_2P_3.1 [41], TRPM8 [42], and K_V_1.2 [43]. Among these, GPR18 has been found to be expressed in the anterior eye and to regulate both intraocular pressure and wound healing [44,45]. Interestingly TRPM8 activation has also been shown to interfere with inflammation induced via TRPV1 in conjunctival epithelial cells [46]. Last, but not least, it should also be noted that degradation of AEA by its major catabolic enzyme FAAH, the expression of which was demonstrated in the current study both in vitro in HCECs and in situ in human corneal epithelium (Figure 1 and Figure 2), results in the production of the pro-inflammatory lipid mediator arachidonic acid (AA). Importantly, AA and its derivatives, e.g., leukotriene B4, are central players in a wide variety of ophthalmic diseases, including cornea-related pathologies. Moreover, their inhibition has recently emerged as a promising, novel therapeutic tool in ocular inflammatory processes. It is possible that, under certain conditions, AEA may be rapidly metabolized to AA and subsequently to other pro-inflammatory mediators, which, for as yet unknown reasons, did not happen in the presence of TRPV1 activators [18,37]. Obviously, with our present understanding of the effects of AEA, it is still not clear exactly which of the above mechanisms could contribute to the pro-inflammatory effects described above; therefore, further studies are invited to unveil the delicate details of the molecular mechanism of the effects of AEA in human corneal epithelium.

Exploiting the endocannabinoid regulation of ocular inflammation appears to be an exciting avenue to explore for possible novel therapies. However, our current results show that caution is warranted when intervening in such a complex and multifaceted system, since, under certain conditions, endocannabinoids might exert pro-inflammatory actions in human corneal epithelial cells instead of the expected anti-inflammatory response.

## 4. Materials and Methods

### 4.1. Materials

AEA was purchased from Cayman Chemical Company (Ann Arbor, MI, USA), p(I:C) was purchased from Invivogen (Invivogen, San Diego, CA, USA). AEA was dissolved in absolute ethanol (Sigma-Aldrich, St. Louis, MO, USA), while p(I:C) was dissolved in nuclease-free water (Invivogen). Vehicle control contained only the solvent at the same ratio as the treated group (1:1000 for both). 

### 4.2. Cell Culturing

HCECs were cultured in a 1:1 mixture of Ham’s F12 and Dulbecco’s modified Eagle’s medium supplemented with 6 (*v/v*)% fetal bovine serum (FBS, Thermo Fisher Scientific, Waltham, MA, USA), 5 ng/mL human epidermal growth factor (Sigma-Aldrich), and MycoZap™ Plus-CL (1:500; Lonza, Basel, Switzerland). The medium was changed every other day, and cells were sub-cultured at 60–70% confluence. Although the antibiotics used in this study can prevent *Mycoplasma* infection, HCEC cultures were regularly tested for *Mycoplasma* contamination using MycoAlert PLUS *Mycoplasma* Detection Kit (Lonza), and every assessment yielded negative results.

### 4.3. UVB Irradiation

HCECs were cultured in a 1:1 mixture of Ham’s F12 and Dulbecco’s modified Eagle’s medium supplemented with serum and antibiotics in Petri dishes (*d* = 35 mm) as described above. The medium was changed to 800 μL Sebomed Basal Medium (Biochrom, Berlin, Germany; a colorless medium that we routinely use in case of UVB-irradiation of epidermal keratinocytes) [47], and cells were exposed to a UVB (Wavelength: 312 nm) irradiation at a dose of 40 mJ/cm^2^. A Bio-Sun microprocessor-controlled UV irradiation system (Wilber Lourmat, Marne-la-Vallée, France) was used for UVB irradiation treatment of the cells. Immediately after the irradiation, the Sebomed Basal Medium was changed to the conventional culture medium of the cells (see above). 

### 4.4. Immunohistofluorescence

Corneal samples were collected from cadaver donors according to guidelines set forth by the local ethical committee (County Government Office Permission No.: IX-R-052/00016-28/2012). Sections were fixed in acetone, permeabilized by 0.1% Triton X-100 in phosphate-buffered saline (PBS; 80.0 g NaCl, 11.6 g Na_2_HPO_4_, 2.0 g KH_2_PO_4_, 2.0 g KCl, dH_2_O to 10 L, all from Sigma-Aldrich), and then incubated with primary antibodies diluted in PBS supplemented with 2% bovine serum albumin (BSA; from Sigma-Aldrich) against FAAH (mouse monoclonal; 1:200), CB_1_ (rabbit monoclonal; 1:250; Abcam, Cambridge, UK, cat. numbers: ab54615 and ab172970, respectively), NAPE-PLD (rabbit polyclonal; 1:1000), MAGL (mouse monoclonal; 1:150; Novus Biologicals, Littleton, CO, cat. numbers: NB110-80070 and NBP2-00735, respectively), cannabinoid receptor type 2 (CB_2_; 1:250), DAGLα (1:250), and DAGLβ (1:250; all mouse monoclonal; Santa Cruz, Heidelberg, Germany, cat. numbers: sc-293188, sc-390409, and sc-514738) at 4 °C overnight. Sections were washed three times and were then incubated with Alexa Fluor 568-conjugated goat-anti-rabbit or Alexa Fluor 555-conjugated donkey-anti-mouse secondary antibodies (cat. numbers: A-11011, A- 31570; Thermo Fisher Scientific; 1:1000; 2 h at room temperature). Sections were washed three times and fixed for 5 min in 1% formaldehyde. Nuclear counterstaining was performed using 4′,6-diamidino-2-phenylindole (DAPI; Sigma-Aldrich) and, after washing, sections were mounted in 10 μL Mowiol (0.1 M Tris-HCl, pH 8.5, 25%(*w/v*) glycerol, and 10% Mowiol 4-88, Hoechst Pharmaceuticals, Frankfurt, Germany). Images were captured with a Zeiss LSM 880 fluorescent microscope (Carl Zeiss AG, Oberkochen, Germany). To ensure that the observed fluorescent signals were not due to nonspecific binding of secondary antibodies, negative controls were obtained by omitting the primary antibody in all cases. 

### 4.5. Immunocytofluorescence

HCECs were cultured on coverslips until 60–70% confluence and fixed in −20 °C acetone for 10 min. Cells were blocked and permeabilized with a mixture of 0.6% Triton X-100 (Sigma-Aldrich) and 1 g/mL BSA in PBS (both from Sigma-Aldrich) for 5 min at room temperature. Cells were probed with the abovementioned primary antibodies against FAAH (1:200), CB_1_ (1:50), NAPE-PLD (1:200), MAGL (1:100), and CB_2_, DAGLα, and DAGLβ (all 1:50) overnight at 4 °C. Following appropriate washing in PBS, coverslips were incubated with Alexa Fluor 488-conjugated goat-anti-mouse and goat-anti-rabbit secondary antibodies (1:1000, cat. number: A-11001 and A32731, Thermo Fisher Scientific) for 1 h at room temperature. Nuclei were counterstained with DAPI (Sigma-Aldrich). To ensure that the observed fluorescent signals were not due to nonspecific binding of secondary antibodies, negative control cells were stained by omitting the primary antibodies. Images were captured with an Olympus Xcellence RT fluorescent microscope (Olympus, Tokyo, Japan).

### 4.6. RNA Isolation, Reverse Transcription, and Quantitative “Real-Time” PCR (Q-PCR) 

RT-qPCR was performed on a LightCycler 480 Instrument II (Roche Life Science, Penzburg, Germany) using the 5′ nuclease assay. Total RNA was isolated using TRIzol (Thermo Fisher Scientific), and DNase treatment was performed according to the manufacturer’s protocol. Then, 1 μg of total RNA was reverse-transcribed into cDNA by using a High Capacity cDNA Kit (Thermo Fisher Scientific). PCR amplification was performed by using the TaqMan assays (IDs: Hs00174092_m1 for interleukin [IL]-1α, Hs00174097_m1 for IL-1β, Hs00985639_m1 for IL-6, Hs00174103_m1 for IL-8) and the TaqMan universal PCR master mix protocol (Thermo Fisher Scientific). As internal controls, transcripts of *18S RNA* or peptidylprolyl isomerase A (PPIA) were determined (assay IDs: Hs03928905_g1 and Hs99999905_m1, respectively). The amount of the abovementioned transcripts was normalized first to the expression of the internal control gene, then to the expression found in the relevant control samples using the 2^−ΔΔCt^ method [48].

### 4.7. Determination of Cytokine Release (ELISA)

Supernatants were collected from HCECs exposed to relevant treatments and were subsequently analyzed for human cytokines using commercially available ELISA kits (IL-6 and IL-8, BD Pharmingen, Franklin Lakes, NJ, USA), according to the manufacturer’s protocols. In brief, plates were coated with capture antibody diluted in coating buffer (0.1 mol/L sodium carbonate, pH 9.5, with 1 mol/L NaOH; Sigma-Aldrich) and incubated overnight at 4 °C. Plates were incubated with Assay Diluent (10% FBS [Thermo Fisher Scientific] in PBS, pH 7.0]) at room temperature for 1 h, and standard and sample dilutions were prepared in assay diluent. Standard and samples were added into appropriate wells and incubated for 2 h at room temperature. After 2 h, working detector (Detection Antibody & SAv-HRP reagent) was added to each well and incubated for 1 h at room temperature. After each step, plates were washed with wash buffer (0.05% Tween-20 [Sigma- Aldrich] in phosphate-buffered saline). After washing, substrate solution (tetramethylbenzidine [Sigma-Aldrich] and hydrogen peroxide in citrate buffer, pH 5.0) was added to each well for 30 min in the dark, followed by stop solution (1 mol/L H_2_SO_4_). Absorbance was measured at 450 nm within 30 min of stopping the reaction using an Envision^®^ 2105 Multimode Plate Reader (PerkinElmer, Waltham, MA, USA). The amount of cytokines in pg/mL was calculated from the relevant standard curves. The experiments were repeated three times using independently collected supernatants.

### 4.8. MTT Assay

Cell viability was assessed by an MTT assay. Cells were plated in 96-well plates (10,000 cells per well density) in quadruplicates and the number of viable cells was determined by measuring the conversion of the tetrazolium salt MTT (3-(4,5-dimethylthiazol-2-yl)-2,5-diphenyltetrazolium bromide; Sigma-Aldrich) to formazan by mitochondrial dehydrogenases. 

### 4.9. Statistical Analysis

Data were analyzed by GraphPad Prism 8.3.1 (GraphPad Software LLC, San Diego, CA, USA) using one-way ANOVA with Bonferroni’s post hoc test (multiple comparisons), and *p* < 0.05 values were regarded as significant differences. Graphs were plotted using Origin Pro Plus 6 software (Microcal, Northampton, MA, USA).

## Data Availability

The data that support the findings of this study are available from the corresponding author upon reasonable request.
